# Evaluating Parkinson's Disease Knowledge Among Academic Healthcare Educators: Findings From a Saudi Arabian Institution

**DOI:** 10.7759/cureus.102473

**Published:** 2026-01-28

**Authors:** Khalid Jambi

**Affiliations:** 1 Clinical Laboratory, Taif University, Taif, SAU

**Keywords:** academic educators, education, faculty development, knowledge gaps, parkinson's disease

## Abstract

Introduction: Parkinson's disease (PD) is a common neurodegenerative condition requiring accurate clinical knowledge for effective patient care. Academic healthcare educators play a key role in preparing future clinicians, yet their PD knowledge has not been well studied. This research assessed PD knowledge among healthcare educators at a Saudi Arabian university.

Methods: A cross-sectional survey was conducted at the College of Applied Medical Sciences, Taif University. Academic staff (N=46), including nurses, radiologists, physiotherapists, and laboratory technologists, completed a validated 20-item questionnaire covering three domains: Diagnosis, Treatment, and Prognosis. Knowledge scores were benchmarked against a model answer, and one-way ANOVA was used to analyze performance differences by profession, academic rank, and degree level.

Results: The cohort demonstrated a moderate to low overall knowledge level, with a mean score of 9.86±2.11 out of 20. Participants performed best in Diagnosis but showed significant deficits in the Treatment domain (3.00±1.3 out of 7). Notably, knowledge gaps were systemic; there were no statistically significant differences in scores based on specialty (p=0.1), academic rank (p=0.348), or educational qualification (p=0.289). Specific misconceptions regarding disease progression rates and stem cell therapy were prevalent across all subgroups.

Conclusions: Academic healthcare educators exhibited uniform knowledge deficits regarding PD, particularly in therapeutic management. The lack of variance by seniority suggests that advanced academic credentials do not guarantee clinical currency in neurodegenerative care. Targeted faculty development initiatives are recommended to prevent the transmission of misconceptions to undergraduate students.

## Introduction

Parkinson's disease (PD) is a progressive neurodegenerative disorder with motor and non-motor manifestations (e.g., hyposmia, sleep behavior disorder), the latter often preceding motor symptoms by over a decade [1]. Surveys of the general public suggest a focus on motor features; for example, while tremor recognition is high, awareness of non-motor features such as sleep disturbances remains lower [2-5]. In contrast, evidence on PD knowledge among specialized audiences (especially the academic educators who shape future clinicians' understanding) is comparatively limited, as most prior work has focused on practicing clinicians rather than those who teach them.

In many medical schools and health-science programs, educators are not purely academic: a proportion hold adjunct or part-time clinical appointments and log limited practice hours across hospitals or clinics. This hybrid role means they both teach PD content and apply it, although not at the volume of full-time clinicians. To address the evidence gap for this audience, we assessed PD knowledge among academic healthcare educators, including nursing, radiology, physiotherapy, and laboratory sciences, at a single college of applied medical sciences in Saudi Arabia using a previously validated questionnaire [6]. We chose this instrument because its content and scoring have been vetted and it enables comparability with earlier clinician-focused work. Our cohort is distinct in that they are not members of the general public but educators (many with some clinical duties) whose instructional responsibilities imply a higher baseline of accurate understanding. By benchmarking responses against a model answer and examining patterns by profession, degree, and academic rank, we aim to identify specific misconceptions within our faculty.

The present study aimed to evaluate PD knowledge among academic educators at the College of Applied Medical Sciences, Taif University. Specifically, we examined whether educators' knowledge levels fall significantly below a set of questions scored at 20 and whether such deficits are uniformly present across subgroups, thereby providing a baseline to guide local faculty development initiatives.

## Materials and methods

Study design and participants

A cross‑sectional survey of academics in the College of Applied Medical Sciences, Taif University, Saudi Arabia, was distributed online in English and was conducted throughout the academic year 2024-2025 (August 2024 to June 2025) after obtaining approval from the institution's Ethics Committee (approval number: HAO-02-T-105; date: May 23, 2024). All participants were informed about the purpose of the study and consented to participate. The cohort included nurses, radiologists, physiotherapists, and laboratory technologists. Demographic information collected included age, gender, profession, level of education (bachelor, master, PhD), and academic rank (assistant professor, associate professor, lecturer, teaching assistant, technician). From the initial cohort of 48 participants, two individuals (one physician and one professor) were excluded to ensure that all reported subgroups met a minimum sample size threshold for statistical comparability and to maintain a homogeneous sample of allied health professionals.

Survey instrument

The instrument was identical to that used in Bhidayasiri et al. [6], which included 20 statements about PD covering three domains (PD diagnosis, treatment, and prognosis). Participants responded "True" or "False" to 19 items, and one item was multiple‑select. English is the official medium of instruction and professional communication at the Medical Field in Saudi Arabia including the institution where this study takes place. Therefore, the English version of the validated instrument was deemed linguistically appropriate for this specific academic cohort without the need for translation.

Scoring

The answers were compared to the model answer used in Bhidayasiri et al. [6]. To quantify participant knowledge, a structured scoring system was developed. The questionnaire was divided into three domains: Diagnosis (eight items), Treatment (seven items), and Prognosis (five items). Correct responses were assigned a score of 1, and incorrect responses were assigned a score of 0. For the single multiple-select question regarding symptoms, partial credit was awarded based on the proportion of correct options selected relative to the total number of correct options available, capped at a maximum of 1 point. The total knowledge score was calculated as the sum of all item scores, ranging from a minimum of 0 to a maximum of 20.

Statistical analysis

Descriptive statistics, including frequencies and percentages, were used to summarize categorical demographic variables (e.g., gender, rank, nationality). Continuous variables, such as knowledge scores, were presented as mean±standard deviation (SD). Normality of the data distribution was assessed using the Shapiro-Wilk test and visual inspection of histograms. To evaluate differences in mean knowledge scores across demographic groups (e.g., academic rank, field of practice), one-way ANOVA was utilized. A p-value of <0.05 was considered statistically significant for all analyses. Analyses were executed in RStudio (Version 4.5.1, Posit PBC, Boston, Massachusetts, United States).

## Results

Participant characteristics are summarized in Tables 1-2 and show representation across nursing, radiology, physiotherapy, and laboratory sciences, with a spread of degree levels and academic ranks.

**Table 1:** Age distribution of the participants

Characteristic	Value
Mean (SD)	37.8 (6.84)
Median	38.0
Range	23.0-56.0

**Table 2:** Sociodemographic and academic characteristics of the participants

Characteristic	N (%)
Gender	
Male	37 (80.4)
Female	9 (19.6)
Level of education	
Bachelor	16 (34.8)
Masters	8 (17.4)
PhD	22 (47.8)
Profession	
Lab technologist	23 (50)
Nurse	10 (21.7)
Radiologist	10 (21.7)
Physiotherapist	3 (6.5)
Academic rank	
Associate professor	6 (13)
Assistant professor	16 (34.8)
Lecturer	12 (26.1)
Teaching assistant	8 (17.4)
Technician	4 (8.7)

**Figure 1:**
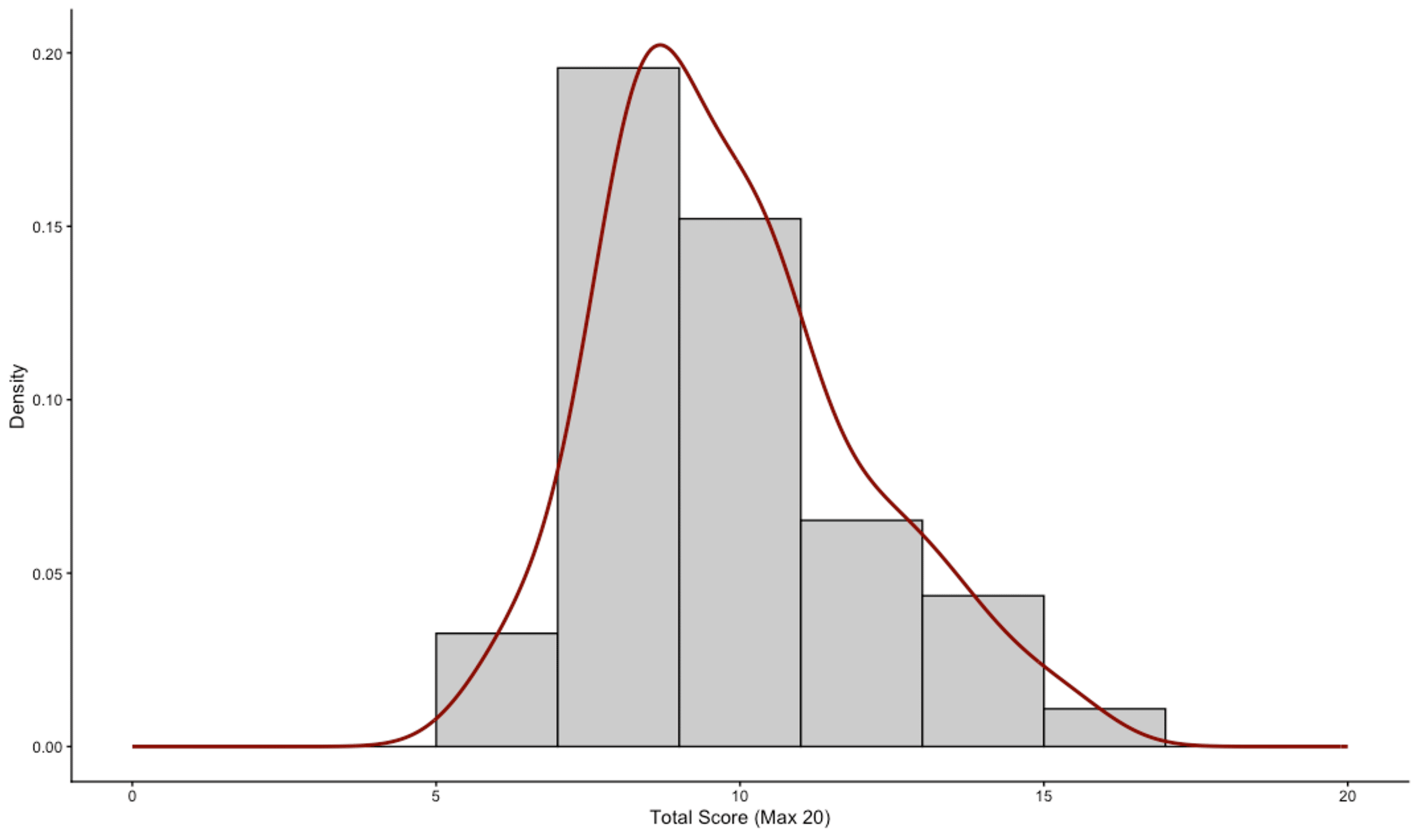
Distribution of total Parkinson's disease knowledge scores among healthcare educators The histogram displays the frequency distribution of total knowledge scores (N=46). The solid red curve represents the density estimation of the data. The distribution approximates normality, indicating a central tendency around the moderate knowledge range.

The overall level of knowledge regarding PD among the participants was found to be moderate to low. The cohort's overall knowledge score ranged from a minimum of 6.0 to a maximum of 15.4, with a mean of 9.86±2.11 SD. The distribution of total scores approximated a normal distribution, as illustrated in Figure 1.

**Figure 2:**
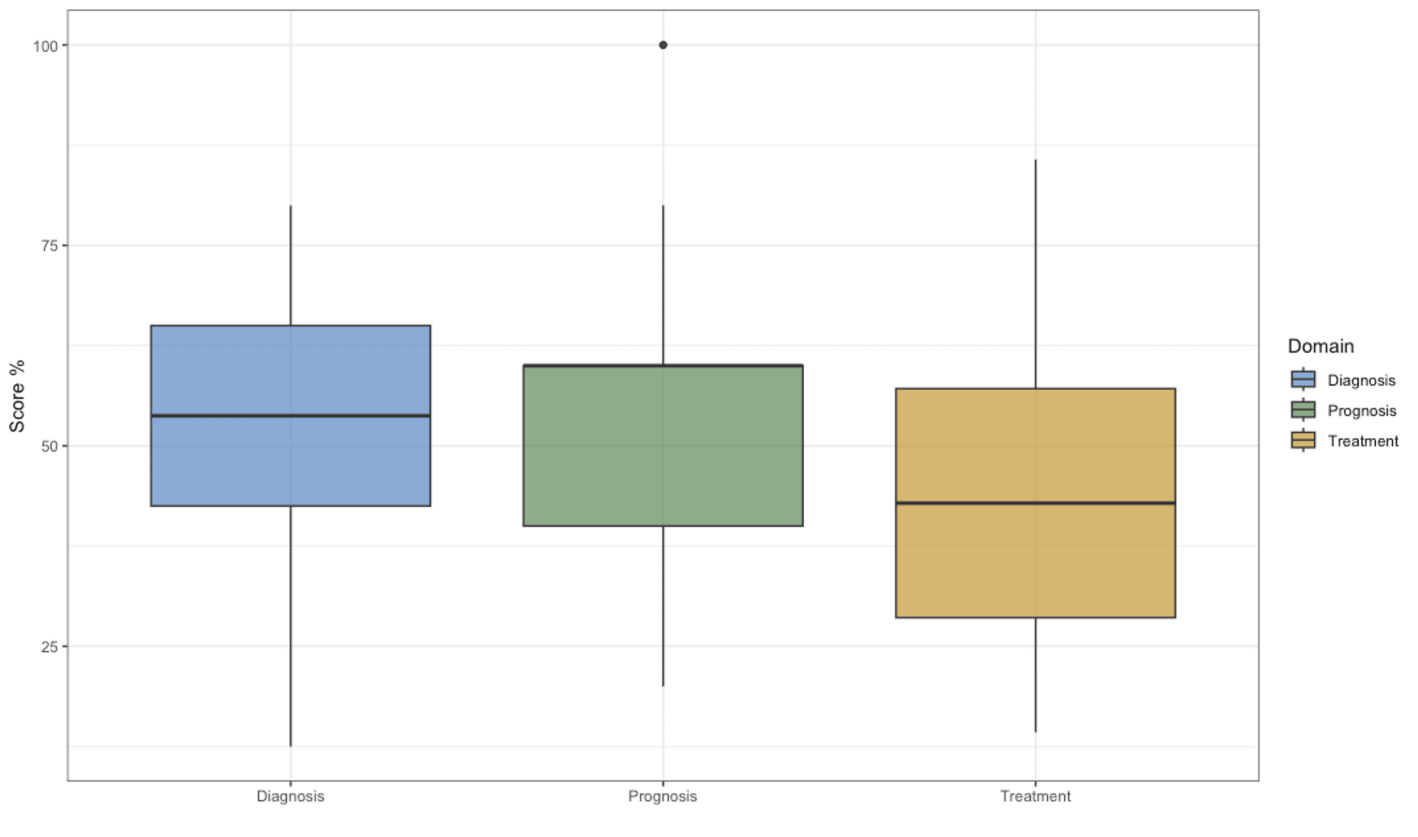
Comparative analysis of knowledge scores across Diagnosis, Treatment, and Prognosis domains Boxplots represent the normalized percentage accuracy for each of the three knowledge domains. The central line within each box indicates the median score, while the box edges represent the interquartile range (25th to 75th percentiles). Whiskers extend to the minimum and maximum values. Participants demonstrated lower proficiency in the Treatment domain compared to Diagnosis.

When knowledge was stratified by domain, participants demonstrated the highest proficiency in Diagnosis, followed by Prognosis, while knowledge regarding Treatment was notably lower. The mean score for the Diagnosis domain was 4.21±1.2 out of 8, whereas the mean score for the Treatment domain was only 3.00±1.3 out of 7. A normalized comparison of percentage accuracy across the three domains is shown in Figure 2, highlighting a specific knowledge deficit in Treatment compared to the other domains.

**Figure 3:**
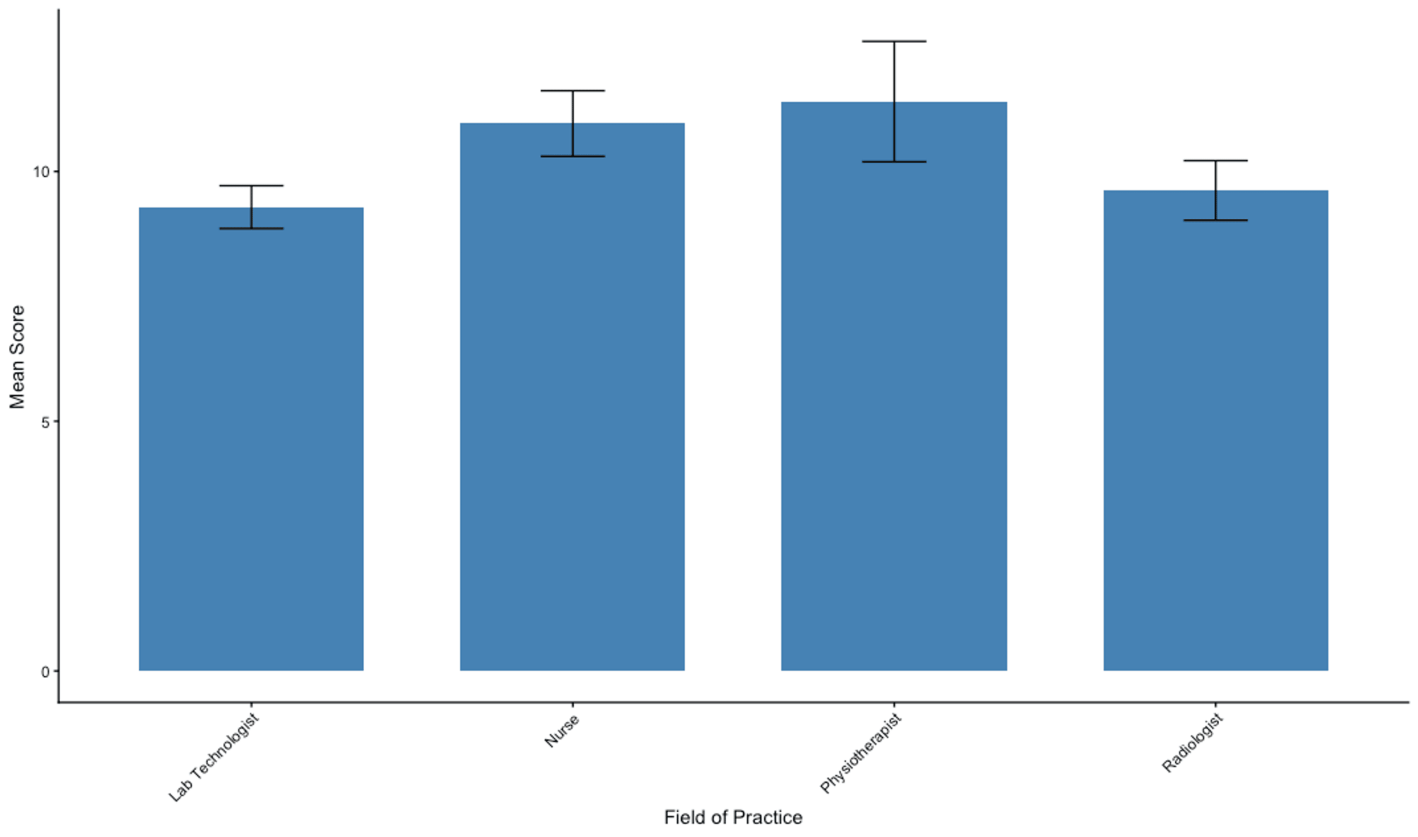
Mean total knowledge scores stratified by field of practice Bars represent the mean total knowledge score (out of 20) for each subgroup. Error bars indicate the standard error of the mean (SEM). No statistically significant differences were observed between groups (p>0.05), suggesting a consistent level of knowledge across different specialties.

To determine if specific demographic variables predicted higher PD knowledge, statistical analyses were performed using one-way ANOVA. Interestingly, the analysis revealed no statistically significant differences in total knowledge scores across the studied demographic groups (p>0.05). There was no significant difference in scores between nurses, physiotherapists, radiologists, and lab technologists (p=0.1), suggesting that knowledge gaps are prevalent across the interdisciplinary fields rather than isolated to a specific specialty (Figure 3).

**Figure 4:**
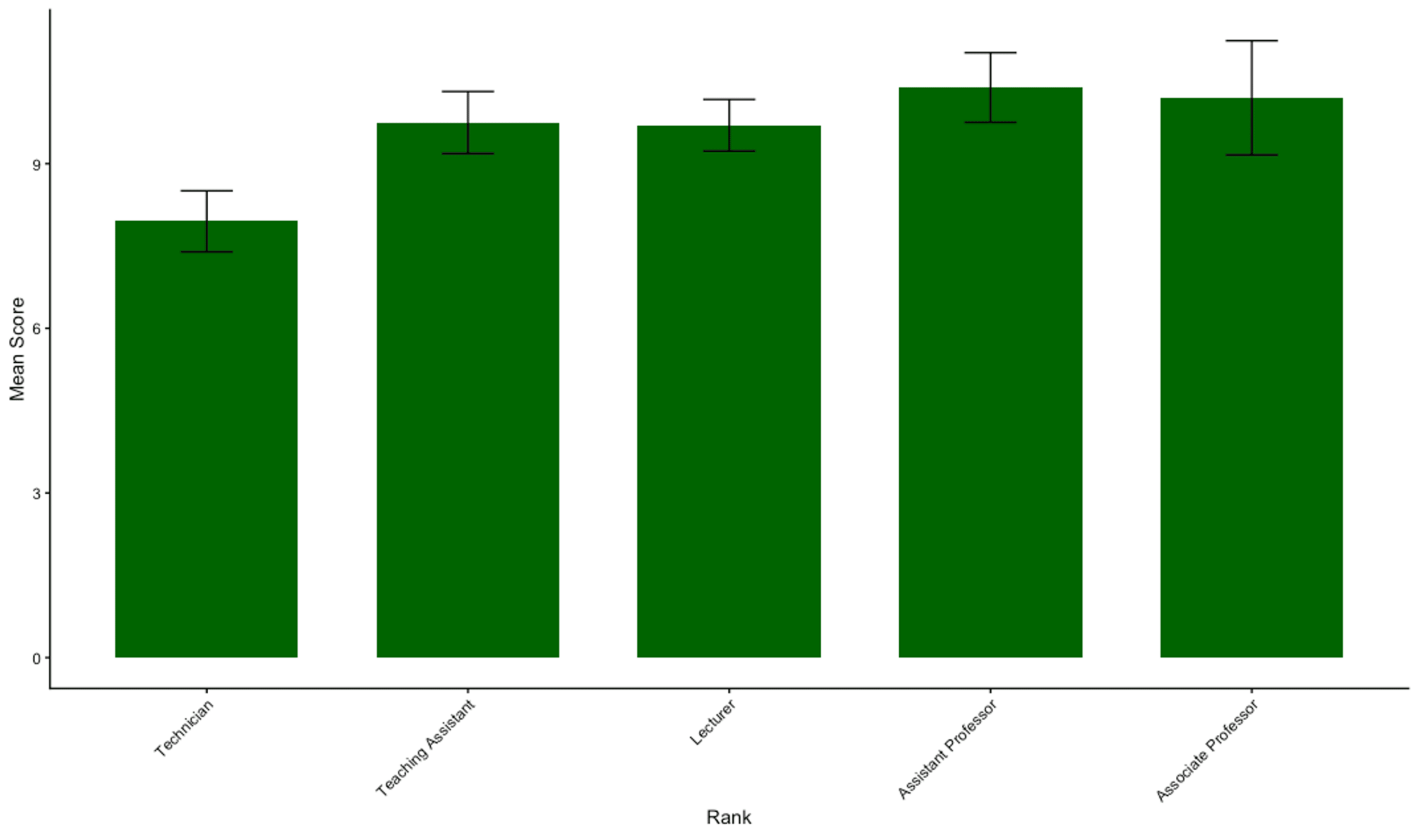
Mean total knowledge scores stratified by ranks Bars represent the mean total knowledge score (out of 20) for each subgroup. Error bars indicate the standard error of the mean (SEM). No statistically significant differences were observed between groups (p>0.05), suggesting a consistent level of knowledge across different ranks.

**Figure 5:**
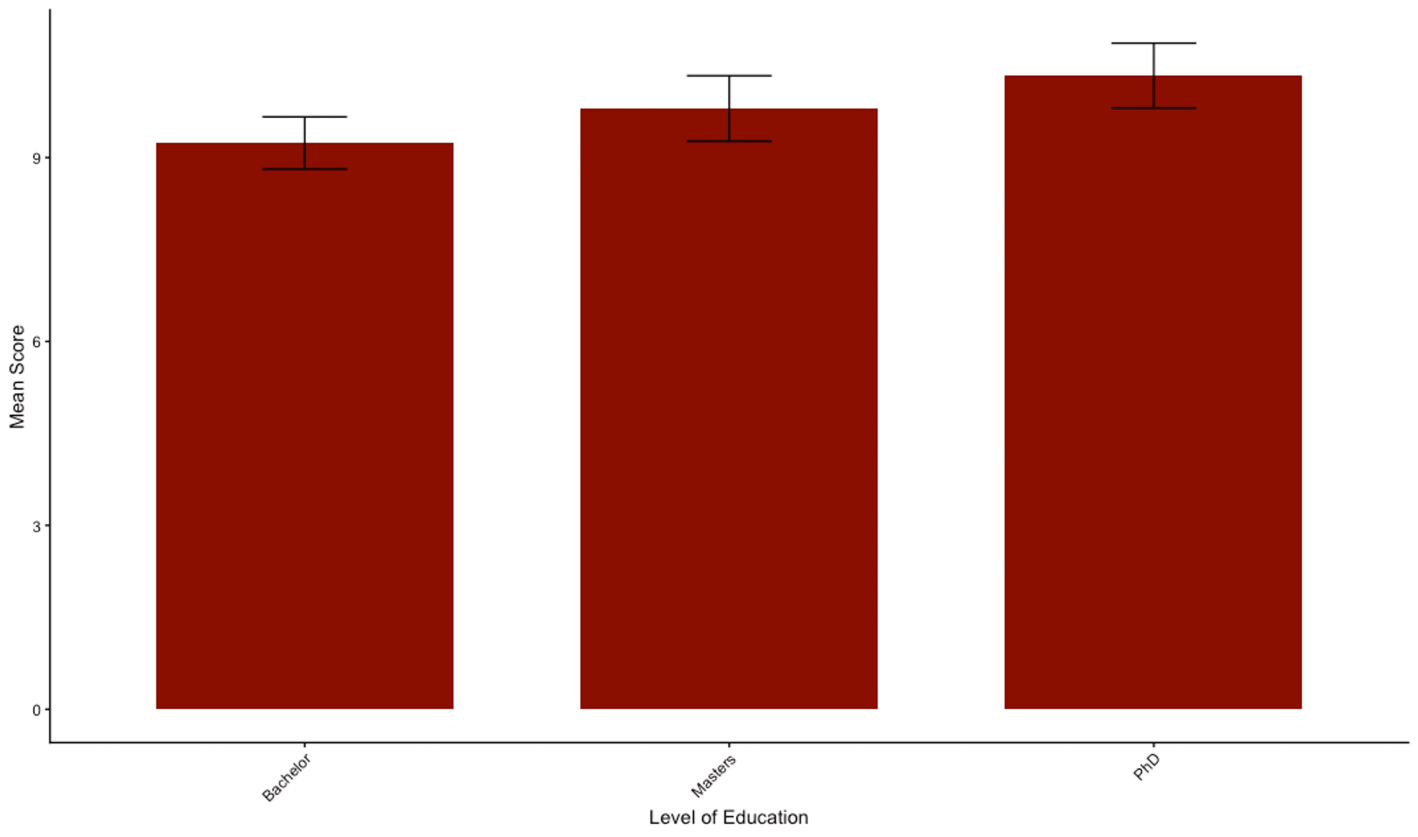
Mean total knowledge scores stratified by level of education Bars represent the mean total knowledge score (out of 20) for each subgroup. Error bars indicate the standard error of the mean (SEM). No statistically significant differences were observed between groups (p>0.05), suggesting a consistent level of knowledge across different level of education.

Moreover, seniority and level of education did not correlate with improved knowledge; no significant performance difference was observed between assistant professors, lecturers, and technicians (p=0.348) and between participants holding a PhD, master's, or bachelor's degrees (p=0.289) (Figures 4-5). 

**Figure 6:**
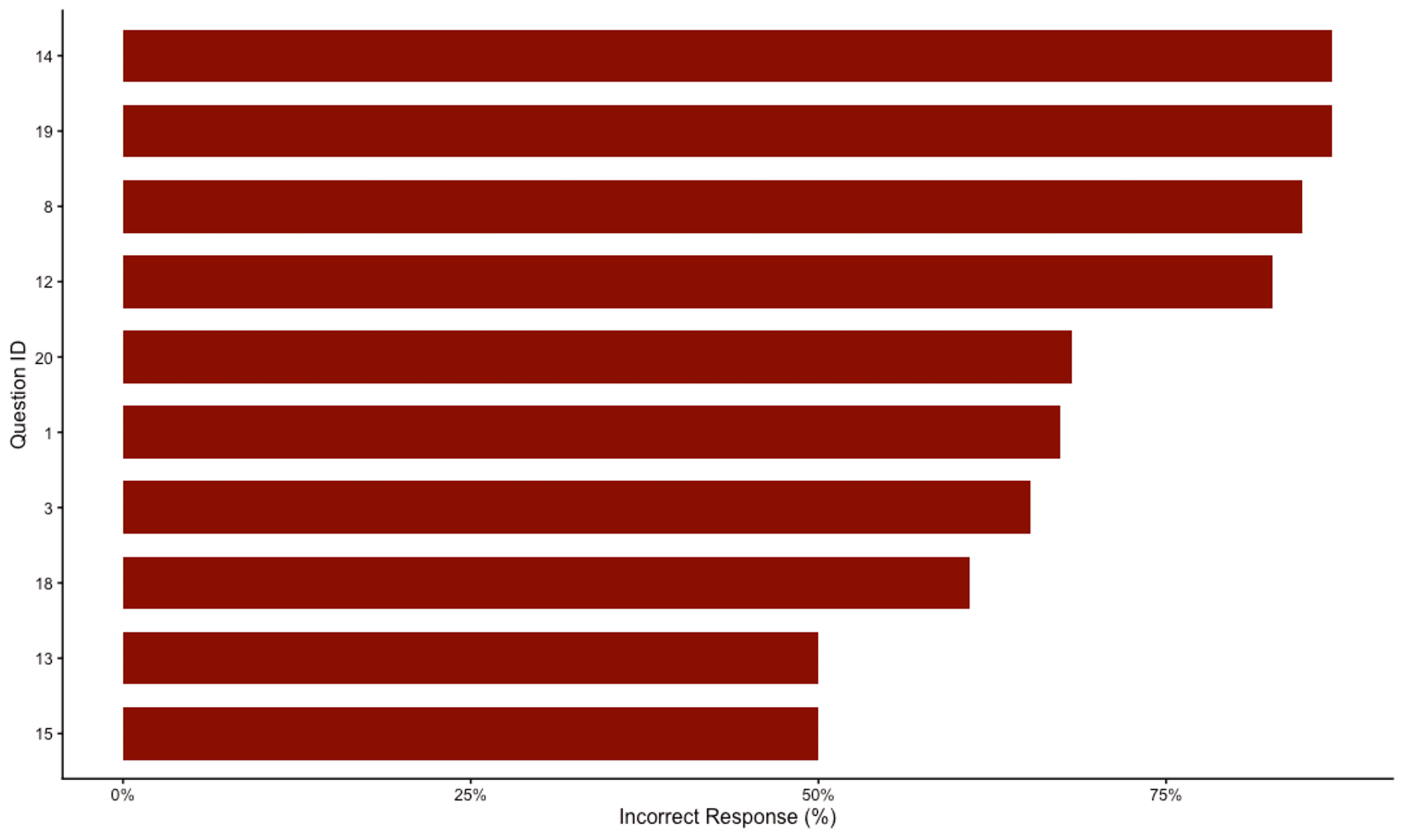
Item analysis identifying the 10 most challenging assessment items The horizontal bar chart ranks the 10 questions with the highest percentage of incorrect responses. Question IDs (Q1, Q14, etc.) correspond to the specific survey items listed in Table 3. The low accuracy rates in these specific items highlight critical knowledge gaps, primarily related to medication side effects and surgical indications.

Item-level analysis identified specific areas of uncertainty among the participants. The questions with the lowest accuracy rates were primarily related to Treatment. As detailed in Table 3 and Figure 6, the three most challenging concepts for the cohort were question 14 (Patients with tremor predominant symptoms progress more rapidly in the degenerative process than those with postural instability and gait problems) followed by question 19 (Current evidence supports the use of stem cell transplantation as a curative treatment of PD) and question 8 (Most of the available drug for PD both treat the symptoms and slow the progression of the disorder) with an incorrect response of 86.96%, 86.96%, and 84.78%, respectively. Conversely, participants demonstrated high accuracy in question 16 (Depression in PD is partly caused by dopamine deficiency) which was correctly answered by 91.3% of respondents.

**Table 3:** Ten most frequently incorrect PD knowledge questions PD: Parkinson's disease

Question ID	Question (area of knowledge)	Incorrect %
14	Patients with tremor predominant symptoms progress more rapidly in the degenerative process than those with postural instability and gait problems (PD Progression).	86.96
19	Current evidence supports the use of stem cell transplantation as a curative treatment of PD (PD Treatment).	86.96
8	Most of the available drug for PD both treat the symptoms and slow the progression of the disorder (PD Treatment).	84.78
12	Dopamine agonists have definite evidence on slowing disease progression (PD Treatment).	82.61
20	Which of the following symptoms of PD do you think often precede tremors and/or bradykinesia and dyskinesias? You may choose all that apply (PD Diagnosis).	68.26
1	Definite diagnosis of PD requires neuroimaging confirmation (PD Diagnosis).	67.39
3	The presence of rest tremor is mandatory for the diagnosis of PD (PD Diagnosis).	65.22
18	Deep brain stimulation surgery can stop PD progression (PD Progression).	60.87
13	PD can be cured by deep brain stimulation surgery (PD Treatment).	50
15	Wheelchair-or bed-bound is inevitable in PD (PD Progression).	50

Taken together, our findings indicate a uniform, widespread knowledge gap in PD knowledge among academic healthcare professionals, identifying specific gaps in treatment protocols that require urgent curricular review. 

## Discussion

This single-institution study reveals that healthcare academics at the College of Applied Medical Sciences at Taif University, regardless of their specific role or seniority, hold significant misconceptions regarding PD. The overall mean score of 9.86 falls substantially below the max score of 20. Crucially, our statistical analysis demonstrated no significant difference in knowledge scores based on academic rank, education level, or field of practice. This lack of variance is a profound finding; it suggests that the knowledge deficit is systemic within the local academic environment rather than being isolated to junior staff or specific disciplines. The fact that assistant professors and PhD holders did not significantly outperform technicians implies that advanced general academic training does not automatically confer specific clinical competency in neurodegenerative disease management.

Our results align with previous reports documenting PD knowledge gaps among practicing clinicians. For instance, a study of Thai medical professionals using the same instrument found that general practitioners and nurses scored substantially lower than neurologists, with misconceptions most frequently clustering around treatment and disease progression [6]. Our cohort mirrored this pattern, with the lowest scores observed in the Treatment domain. This specific deficit likely reflects the increasing complexity of PD pharmacotherapy and the nuance required to manage levodopa-induced complications, which are challenging to master without frequent clinical exposure [7]. Similarly, studies assessing nursing staff in other regions have identified critical deficits in understanding medication timing and symptom management [8]. The consistency of these findings across both clinical and academic cohorts highlights that these gaps are not limited to patient‑facing roles but extend into the academic settings where the next generation of professionals is being trained.

Furthermore, the challenge of maintaining faculty knowledge is not unique to PD. Similar deficits have been documented among nursing faculty regarding Alzheimer’s disease and notably among health educators in Saudi Arabia regarding diabetes mellitus [9,10]. Our finding that professional rank was not a predictor of knowledge reinforces the conclusion of these previous studies: ensuring academic educators remain current on complex, rapidly evolving diseases is a broad challenge in health sciences education, particularly for faculty who may have limited ongoing clinical practice.

The implications of these findings are considerable. Educators act as the primary gatekeepers of knowledge, and misconceptions at the teaching level risk being transmitted directly to students, potentially leading to incomplete or biased clinical training. This is of particular concern given that undergraduate nursing and allied health students already report low confidence and knowledge regarding the care of neurodegenerative diseases [11]. The specific weakness we identified in the Treatment domain mirrors broader challenges in PD education, where the nuances of advanced therapeutics and non-motor symptoms are often overshadowed by a simplified focus on cardinal motor features [12,13].

A key strength of this study is its use of a validated instrument, which allowed for a standardized assessment of knowledge. However, the study has important limitations. The primary limitation is its single-institution design and modest sample size (N=46), which prevents the generalization of these findings to all healthcare educators in Saudi Arabia. Rather, this study should be viewed as a signal of a potential systemic issue warranting a larger, multi-center investigation. While educators need not match the specialized expertise of neurologists, their fundamental role in shaping curricula necessitates a higher baseline of accurate, contemporary understanding than was observed here.

Addressing these uniform misconceptions will require targeted, evidence-based interventions. Since the deficit is present across all ranks, interactive faculty development workshops and interprofessional seminars are recommended [14]. The experience in the Lao People's Democratic Republic reinforces that PD-specific, practical training can yield rapid, measurable gains in knowledge and should be prioritized within continuing medical education for academic staff [15].

## Conclusions

This study identifies a uniform, system-wide gap in PD knowledge among allied health academics, characterized by marked misconceptions in treatment and disease progression. The observation that knowledge scores did not differ significantly between assistant professors, PhD holders, and technical staff is a profound finding; it implies that general academic advancement does not automatically confer specific clinical competency in complex neurodegenerative diseases. Because educators act as the primary gatekeepers of knowledge, these unaddressed gaps pose a risk of perpetuating incomplete or biased clinical understanding in the next generation of healthcare professionals. While these findings are limited by the single-institution design and sample size, they signal a potential curricular blind spot. To address this, we recommend inter-professional faculty development workshops. Ensuring that educators remain current with evolving PD pharmacotherapy and management strategies is essential to bridging the gap between academic instruction and clinical reality.
